# Climate warming promotes pesticide resistance through expanding overwintering range of a global pest

**DOI:** 10.1038/s41467-021-25505-7

**Published:** 2021-09-09

**Authors:** Chun-Sen Ma, Wei Zhang, Yu Peng, Fei Zhao, Xiang-Qian Chang, Kun Xing, Liang Zhu, Gang Ma, He-Ping Yang, Volker H. W. Rudolf

**Affiliations:** 1 Climate Change Biology Research Group, State Key Laboratory for Biology of Plant Diseases and Insect Pests, Institute of Plant Protection, Chinese Academy of Agricultural Sciences, Beijing, China; 2 College of Plant Protection, Shanxi Agricultural University, Shanxi, China; 3 Hubei Province Key Laboratory for Crop Diseases, Insect Pests and Weeds Control, Institute of Plant Protection & Soil Science, Hubei Academy of Agricultural Sciences, Wuhan, China; 4 National Meteorological Information Centre, Beijing, China; 5 BioSciences, Rice University, Houston, TX USA

**Keywords:** Agroecology, Climate-change ecology, Ecological modelling

## Abstract

Climate change has the potential to change the distribution of pests globally and their resistance to pesticides, thereby threatening global food security in the 21st century. However, predicting where these changes occur and how they will influence current pest control efforts is a challenge. Using experimentally parameterised and field-tested models, we show that climate change over the past 50 years increased the overwintering range of a global agricultural insect pest, the diamondback moth (*Plutella xylostella*), by ~2.4 million km^2^ worldwide. Our analysis of global data sets revealed that pesticide resistance levels are linked to the species’ overwintering range: mean pesticide resistance was 158 times higher in overwintering sites compared to sites with only seasonal occurrence. By facilitating local persistence all year round, climate change can promote and expand pesticide resistance of this destructive species globally. These ecological and evolutionary changes would severely impede effectiveness of current pest control efforts and potentially cause large economic losses.

## Abstract (web summary)

Climate-driven range shifts may affect pesticide resistance. Here, the authors analyse experimentally parameterised and field-tested models to show that a cosmopolitan insect pest, the diamondback moth, is acquiring resistance against local pesticides through expanding overwintering range.

## Introduction

Anthropogenic climate change increases crop losses by pests^1^, and extensive pesticide applications promote pesticide resistance^2^, thereby threatening global food security and food safety in the 21st century^1,3^. Specifically, climate change has expanded the geographic range of many pests in regions that have traditionally experienced low pest risk^4^. At the same time, pesticide usage and pesticide resistance have increased in these regions^5,6^. While these patterns appear linked, climate-mediated range shift and pesticide resistance are traditionally examined independently of each other. Consequently, it is still unknown how changes in pest distribution will affect the development and distribution of pesticide resistance under future climate change scenarios.

To understand the connection between range shifts and pesticide resistance first requires a detailed understanding of how climate change will alter the geographic range of pest species. While much progress has been made in documenting shifts in pest distributions in response to climate change^4^, predicting how distributions will change in the future is challenging. Such predictions require mechanistic models that identify first which aspect of climate change is altering the distribution of pest species^7,8^. In the search of mechanisms, cold temperatures have emerged as a key factor limiting the spread and range of insect species^9,10^; species need to survive cold stress so that growth, development, and reproduction can resume when conditions become more favourable. Consequently, many pest species undergo annual cycles of seasonal migrations^11^: they start spreading from warm overwintering regions into colder regions in late spring and early summer, but disappear in colder regions during fall and winter. Depending on the local conditions, pest species are therefore either able to persist locally all year round (permanent), or they are only present as seasonal (transient) visitors.

The difference in permanent vs. transient occurrence is crucial because it determines population growth rates (and thus pest abundance) and damage periods of pests at a given site. Importantly, the same factor may also determine the potential for pests to evolve pesticide resistance. In locations with permanent populations, pests are often repeatedly exposed to the same pesticide, allowing resistant individuals to increase each year^12^. Eventually, this may lead to local adaptation and pesticide resistance accumulation^13,14^. In contrast, with seasonal (transient) occupancy pests are only exposed to local conditions including pesticides for part of the year^15,16^. This seasonal exposure can potentially lead to a short-term (within year) increase in resistance over time if pests have multiple generations within a given season^17^. However, long-term local adaptation to a unique condition (e.g. pesticide) in a given location is strongly restricted in transient populations, because locally adapted resistant phenotypes die in cold winter temperatures (which can further be exacerbated by resistance-associated fitness (e.g. survival) costs^18^) or emigrate at the end of the season^19^, while colonizers in the next seasons come from different (warmer) locations^20^. If climate change expands the conditions that allow a pest to persist locally (change from transient to permanent), this could concurrently change a pest’s potential to develop resistance against locally employed pesticides. However, predicting where such a climate-mediated increase in resistance occurs should verify the link between overwintering range of pests and their resistance to pesticides.

Here we tested how increasing winter temperatures affect the range limits and pesticide resistance of a global pest, the diamondback moth (*Plutella xylostella*). This moth originated from South America and spread to all other continents^21^. It now ranges from tropic to temperate zones, causing economic loss as high as US$ 4–5 billion per year^22^, making it the most destructive pest of cruciferous crops around the world^23^. The species is also famous for its strong resistance to over 97 different insecticide active ingredients^24^. Importantly, it can only overwinter in warm areas^25^, from which it quickly migrates far north during the growing season, damaging local crops^26^. Although the acute thermal limits (e.g. lower lethal temperature or supper cooling point) have been measured previously^27–31^, they cannot explain the extensive winter mortality at temperatures much higher than its lower thermal limits^32–34^. By analysing experimentally parameterised and field-tested models, we show that climate change over the past 50 years increased the overwintering range of this pest by ~2.4 million km^2^ worldwide. The meta-analysis of global data sets reveals that pesticide resistance levels are linked to the species’ overwintering range. By facilitating local persistence all year round, climate change can promote and expand pesticide resistance of this destructive species globally.

## Results and discussion

### Winter survival model

**Fig. 1:**
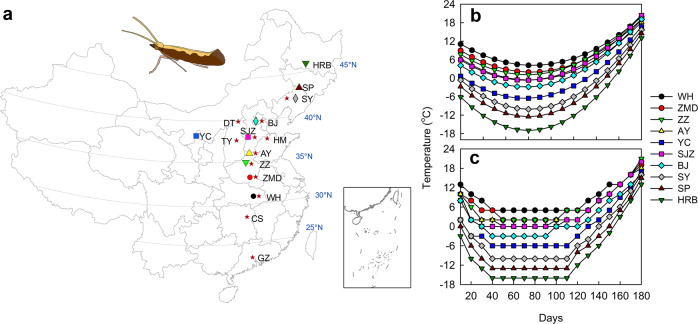
Winter survival experiment for the diamondback moth. **a** Geographic sites selected for the winter survival experiments under laboratory (different symbols) in 2011 and field conditions (stars) in winter (November–April) of 2008–2013. **b** Polynomial curves fitted to the daily mean temperatures of the 10 sites during the winter of 1966–2010 for the laboratory experiments. **c** Simulated winter temperatures representing each of the 10 sites in panel (**b**). The *x*-axis represents the number of days since November 1st and the *y*-axis indicates the mean temperature for a given 10-day interval for panel (**b**). HRB Harbin, SP Siping, SY Shenyang, DT Datong, BJ Beijing, YC Yinchuan, SJZ Shijiazhuang, TY Taiyuan, HM Huimin, AY Anyang, ZZ Zhengzhou, ZMD Zhumadian, WH Wuhan, CS Changsha, GZ Guangzhou. Source data are provided as a Source data file.

**Fig. 2:**
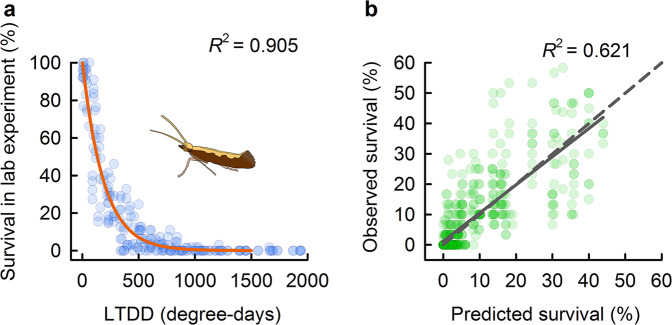
Optimal winter survival model and field validation. **a** Optimal winter survival model of diamondback moth with low-temperature degree-days (LTDD) (*n* = 220). **b** Relationship between model predictions vs. field observations of winter survival (*n* = 556). *R*^2^ indicates the variability explained by the regression model. Blue and green circles represent the survival rates of diamondback moth in laboratory experiments and field investigations, respectively. The red line represents model predictions and the grey solid line represents the linear regression between LTDD model predicted and observed survival under natural conditions across geographically distinct sites (grey dashed line indicates the 1:1 reference line). Source data are provided as a Source data file.

To examine how climate change will affect the distribution and resistance of this species, we first developed and field-tested a mechanistic and predictive framework for winter survival. The model was developed using a set of survival experiments under controlled environmental conditions in the laboratory that exposed 13,200 individuals to 10 temperature regimes representing different geographic sites spread across the range of the diamondback moth in China (Fig. 1, Supplementary Table 1). Fitting alternative mechanistic models to the data across these temperature regimes indicated that 90.5% of observed survival pattern could be explained with an exponential survival model based on the number of degree-days individuals experienced below a threshold of 11.0 °C where pupa and larva suffered reduced survival rates^32^ (= low-temperature degree-days (LTDD)) (Fig. 2a, Supplementary Fig. 1, Supplementary Table 2). Given that survival decreased well before the temperatures dropped to the supercooling point^33,34^, this result indicates that chronic effects of chilling (rather than freezing at extremely low temperatures) are the main thermal drivers that determine winter survival in this pest species^35^. The chronic effect of chilling could be explained by the accumulation of cold injury related to poor ion homoeostasis at low temperatures^35^.

Next, we tested the ability of the model to predict survival under variable natural conditions, by confronting the model predictions derived from our laboratory experiments with survival patterns in the field. Specifically, we conducted separate large-scale field experiments that exposed larvae, pupae or adults to two types of overwintering conditions (standing plant vs. post-harvest conditions) at 12 geographic sites along a latitudinal temperature gradient in China (Fig. 1a, Supplementary Table 1). Consistent with laboratory results, we found that the exponential survival model based on LTDD best-described overwintering survival (Supplementary Table 3, Fig. 2), predicting >62.1% of survival (Fig. 2b). This high level of predictive power is particularly remarkable given the highly variable environmental conditions across field sites and years. It also verifies that the mechanistic LTDD model can reliably predict survival patterns under a wide range of natural conditions.

### Winter warming increases overwintering range globally

**Fig. 3:**
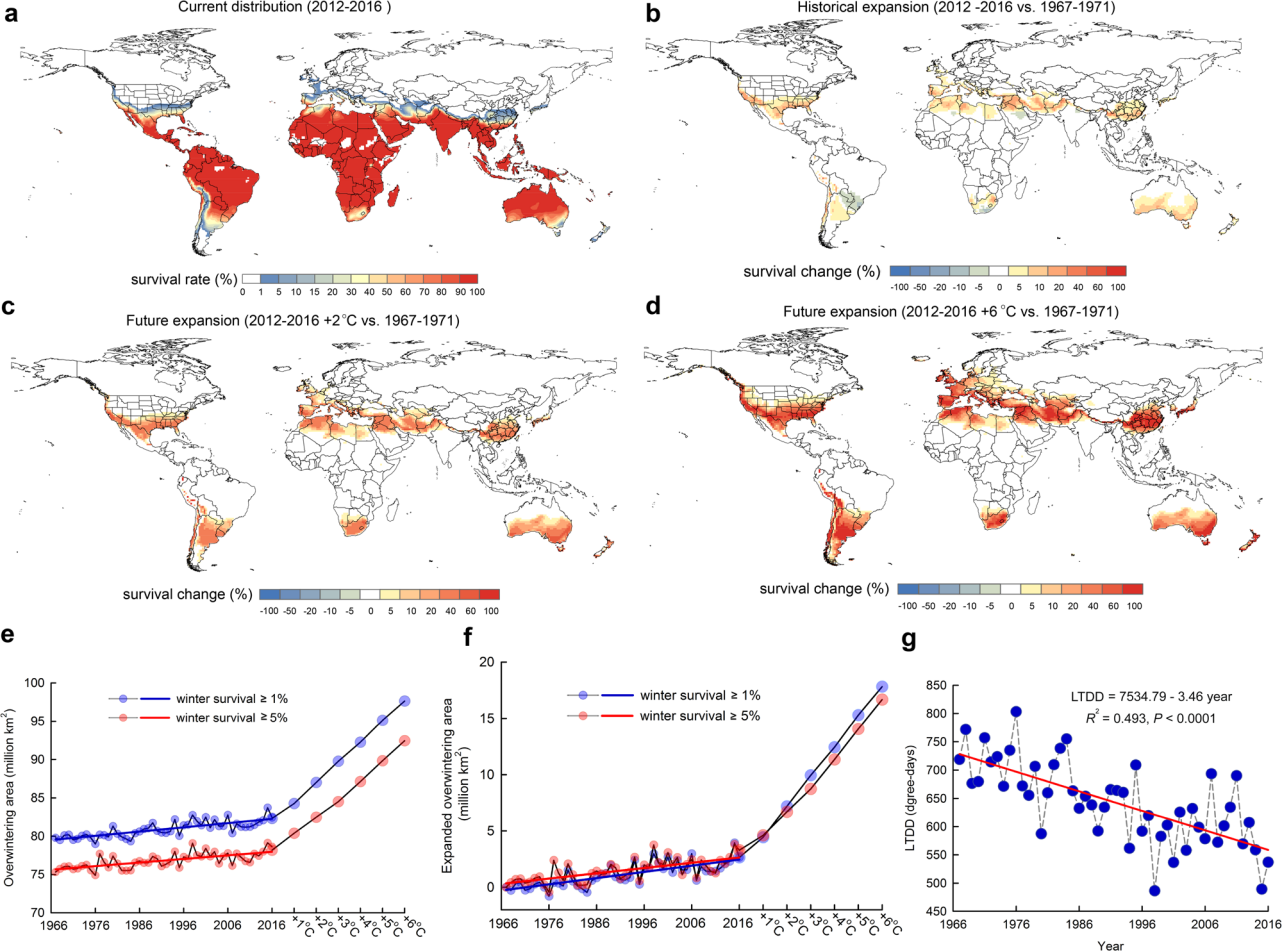
Predicted global overwintering survival of the diamondback moth. **a** Mean distribution of winter survival in the present 5 years. **b** Changes in winter survival over the past 50 years. **c**, **d** Changes in winter survival for future climate scenarios with a predicted +2 °C or +6 °C increase in mean temperatures. **e** Predicted global overwintering land area, and **f** expanded overwintering area at given past and possible future warming scenarios. **g** Global annual dynamics of low-temperature degree-days (LTDD) in the overwintering marginal belt. In the tendency line, *R*^2^ indicates the variability explained by the regression line. *P* value was calculated using two-sided Fisher’s test. All predictions are based on the field validated LTDD-dependent survival model. Source data are provided as a Source data file.

To examine changes in the overwintering range of this pest species under different climatic conditions, we combined the validated survival LTDD model with global temperature data from the past 50 years and future climate scenarios. Besides making biological sense, the LTDD metric has the additional advantage that it can easily be obtained from past climate data and future scenarios, which allowed us to examine how the overwintering potential of this species changes at a global scale. Our analysis revealed that the overwintering range of the diamondback moth (Fig. 3a) has already expanded globally, and will continue to expand under future climate change scenarios (Fig. 3e). Analysis of historical climate data indicates that increasing winter temperatures in the past 50 years^36^ have reduced the degree-days below the critical survival threshold globally (Fig. 3g). Consequently, the overwintering range of the diamondback moth (winter survival ≥5%) has already expanded by ~2.4 million km^2^ over the past 50 years (1967–1971 to 2012–2016) in the United States, Europe, China, Japan and other regions (Fig. 3b, f). This expansion rate appears to increase continuously over time, but a longer time series is needed to confirm this linear trend.

Furthermore, our simulations predict that the potential overwintering land area will continue to expand globally at an average rate of 2.2 million km^2^ for every 1 °C increase in mean global temperature (Fig. 3f). Given that current climate change models predict a temperature increase by 2–6 °C in the next 100–150 years^37^, the model predicts a corresponding increase from 6.7 million km^2^ in the best case scenario (with +2 °C) (Fig. 3c, f) to 16.7 million km^2^ in the worst-case scenario (with +6 °C) (Fig. 3d, f). This trend was qualitatively and quantitatively the same when we looked at the expansion of potential overwintering sites (≥1% survival) or permanent overwintering sites (≥5% survival) (Fig. 3f). This climate-mediated expansion of the overwintering range is predicted to occur across the broad marginal belt, thus affecting agricultural important regions such as Europe, China, Japan, the United States, South America, Africa and Australia (Fig. 3c, d). Given that the central range of this species is in the tropics, it is unlikely that future climate warming scenarios will lead to range contractions in the next 100–150 years.

Predicting how climate change will affect species distributions is a key challenge in the Anthropocene^38^. Traditionally, studies often extrapolate existing distribution patterns to make first predictions about future climate change effects^39^. While these studies have provided valuable first insights, the underlying approach has been increasingly criticized^40,41^, because they often cannot directly infer the conditions and mechanisms that ultimately limit the fundamental niche of a species. To avoid these shortcomings, we used a mechanistic model derived from controlled laboratory and field experiments that identified key factors that limit a fundamental physiological and demographic process (winter survival) of the focal species. Similar approaches could easily be applied to a wide range of other taxa.

When interpreting our model results, it is important to keep in mind that winter survival and overwintering range are not only determined by winter temperatures and host plant availability, but also likely influenced by other climatic and biotic factors such as rainfall or snowfall, natural enemies and other pests that are not included in our current model. Dispersal is unlikely to limit the distribution of this species, given its high dispersal ability and distance each season^26^. Similarly, because it is an agricultural pest, competitors and predators are unlikely to exclude it from a location, although they likely influence local abundances. Given that fluctuating winter temperatures can result in extremely cold events, incorporating acute cold tolerance (the critical thermal minimum (CT_min_) or supercooling points) in the model could potentially increase the confidence of prediction, but requires reliable daily extreme temperatures across sites. Note, however, that fluctuating amplitudes in the microhabitats such as standing plants and debris are typically smaller than air temperatures, buffering against extreme temperatures compared to air conditions. Behavioural thermal regulation in heterogeneous microclimates could potentially also buffer temperature extremes^42–44^, and thus impact pest distribution, but requires again fine scale data on temporal variation. Accounting for these and other factors could be important, especially for predictions at much finer geographic resolution. Until these relationships are firmly established, our model provides a default set of expectations for the range limits of this pest species and how they will be influenced by climate change.

### Overwintering area expansion increases pesticide resistance

**Fig. 4:**
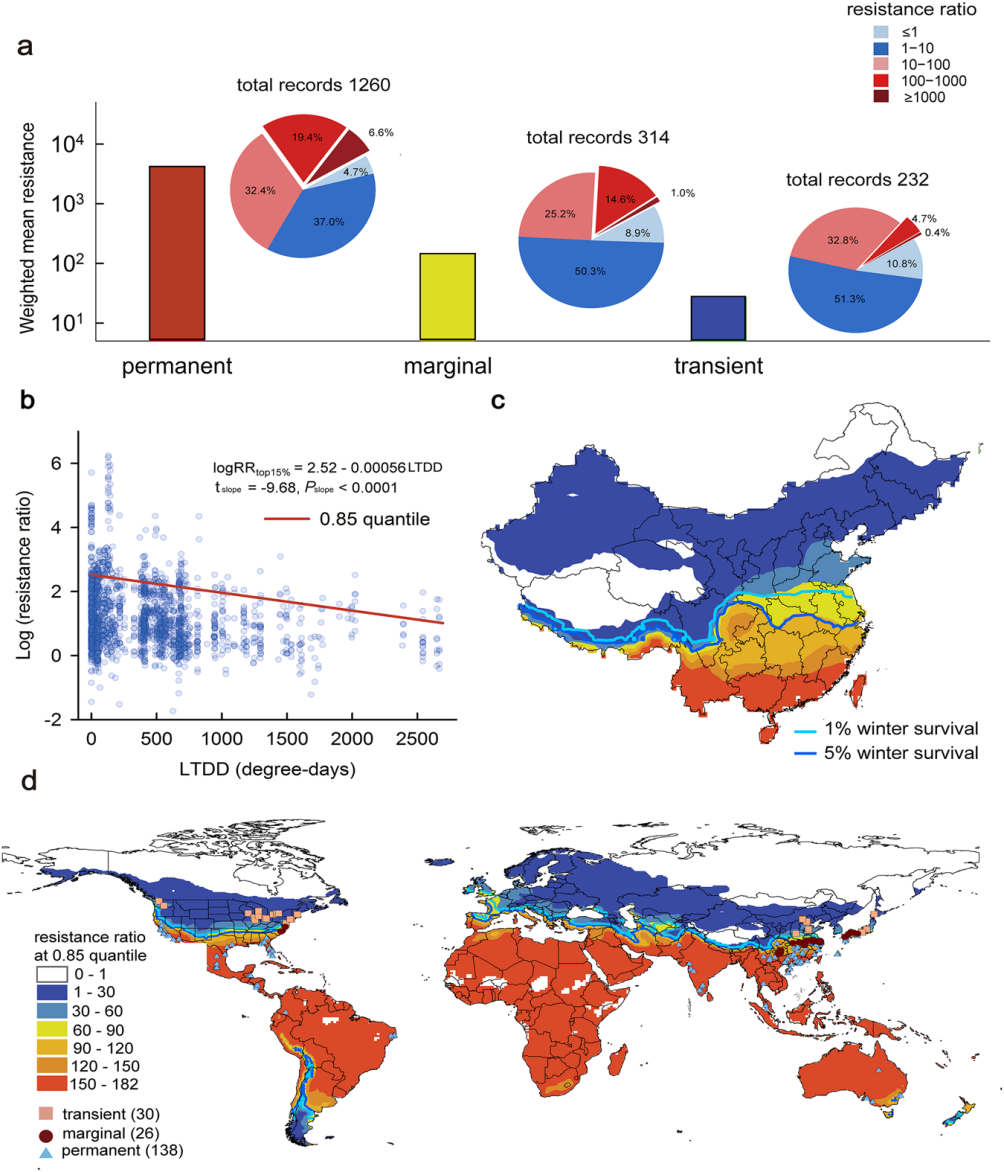
Pesticide resistance of the diamondback moth. **a** Weighted mean resistance (ratio) and relative frequencies of different resistance levels (percentages in the pie charts) in the permanent, marginal and transient sites. **b** Relationship between the top 15% (0.85 quantile) resistance ratio (log_10_ transformed) and low-temperature degree-days (LTDD) (*n* = 1806). The significance of the slope (*P* value) in the quantile regression was tested by two-sided *t*-test. **c** Predicted distribution of the top 15% pesticide resistances in China. **d** Global distribution of sampling sites for pesticide resistance tests used in meta-analysis and the predicted geographic distribution of the top 15% pesticide resistance. Source data are provided as a Source data file.

To examine how the change in overwintering range might affect pesticide resistance, we conducted a meta-analysis based on 1806 published global records. This analysis revealed that pesticide resistance of the diamondback moth is significantly linked to its overwintering type (permanent, marginal, non-overwintering) at a given site (*χ*^2^ = 7.354, *P* = 0.0253), even after incorporating climate conditions during the growing season (i.e. degree-days of effective temperatures for development (ETDD)), pesticide variety, and the interaction effects (Table 1), which are well known to contribute the resistance levels. Mean resistance in the permanent overwintering sites was 158 times higher than that in the non-overwintering (transient) sites and five times higher than that in the marginal belt (Fig. 4a). Furthermore, high resistance (resistance ratio ≥100) occurred four times more frequently in permanent sites (26.0%) than in non-overwintering sites (5.2%) (Fig. 4a). In pest control, practitioners are especially concerned about regional high levels of pesticide resistance. Thus, we built a model to calculate the top 15% resistance level based on LTDD (Fig. 4b) and thereby predict the global distribution of high resistance level in China and the world (Fig. 4c, d).

**Table 1:** Linear mixed model for the effects of overwintering type, pesticide variety and degree-days of effective temperatures (ETDD) during the growing season on weighted pesticide resistance level (weighted logRR) using Chi-square test.

Source	*χ*^2^	*df*	*P*
overwintering type	7.354	2	0.0253
pesticide variety	547.642	14	<0.0001
ETDD	4.515	1	0.0336
pesticide variety*overwintering type	78.825	27	<0.0001
ETDD*pesticide variety	151.316	14	<0.0001

Note: The fixed-effect matrix in the model with the interaction of 3 factors is rank deficient, which leads to failing in fitting the model.

While consistent with theoretical expectations, the dramatic difference in pesticide resistance between permanent and transient overwintering sites is particularly striking, given that pesticide resistance can be influenced by other factors. So far, resistances to 97 different active ingredients of pesticides have been found in the diamondback moth worldwide^24^. Different pesticides and management practices across sites likely result in different levels of selection and/or resistance patterns that could have reduced our power to detect a general pattern across sites. Furthermore, migration may muddle resistance patterns due to the frequent gene exchange in non-overwintering areas that could either promote (the same variety of pesticide between emigrated area and immigrated area)^45^ or reduce the resistance (the different variety of pesticides between emigrated area and immigrated area)^46^. These factors likely account for a good portion of the observed variation in pesticide resistance and could potentially reduce the effect of winter survival on the evolution of pesticide resistance. Yet, we still found a clear difference in pesticide resistance in overwintering vs. non-overwintering sites. Importantly, this pattern persisted even after accounting for differences of temperatures in other seasons (effective degree-days) which are linked to generation time, pesticide application, and general selection strength^47^. Overall, these results indicate that climate warming can be an important driver of pesticide resistance when it expands the conditions that allow a pest to persist locally (change from transient to permanent). Based on our model predictions (Fig. 4b), this would especially affect the pesticide resistance of current “marginal” overwintering regions, such as the US, Mediterranean countries, China and Japan (Fig. 4c, d).

Ongoing climate warming is expected to precipitate a range of possible evolutionary changes. Previous studies have typically focused on evolution in temperature-related traits, such as the evolution of thermal performance adapt to higher temperatures^48,49^. Here we discovered that warming could also promote the evolution of resistance to pesticides. These results demonstrate that climate change can result in indirect evolutionary changes, i.e. changes in traits that are not directly linked to changes in climatic conditions. Although the exact underlying ecological mechanisms are not fully understood^47^, several non-exclusive pathways are likely. Pesticide resistance is usually associated with resistance alleles, which potentially increase the metabolic capability of detoxificative systems^50^ or/and reduce the sensitivity of target sites to pesticides^51^. Therefore, any ecological factors that could increase the frequency of the resistance alleles in a population or contribute to maintaining resistance alleles may accelerate the evolution of pesticide resistance. Warming may increase the selection pressure to resistance alleles by more pesticide application^52,53^, due to more abundant population and longer damage of pests in warmer growing season^54^. Warming can also shorten the generation time of pests^55^ and thereby speed up the rate of resistance evolution^56^. However, with cold winters, populations either die^35^ (especially for the resistant populations which are usually more vulnerable to extremely low temperatures due to fitness costs, e.g. survival reduction^18^) or migrate to warmer areas^11^. As a consequence, the spring population at a given site will most probably be a mixed population immigrated from multiple sites^20^ where different types of pesticides are used, resulting in either the loss of pesticide resistance, or at least slow evolution of resistance in a mixed population where the frequency of locally adapted individuals will be less^57^. In contrast, winter warming can expand the overwintering area, allowing the local population to persist^58^ and thus quickly accumulate resistance alleles across seasons in a given site. The interaction between increased selection for resistance and maintenance of resistance (increased winter survival) under global warming may therefore lead to rapid evolution of pesticide resistance.

Many of the most notorious agricultural pests occur in warm regions throughout the year and migrate to non-overwintering regions^11^ and cause rapid and destructive seasonal damage, including many species of armyworms, planthoppers, leafrollers and aphids. A wide range of species in different insect orders, including Coleoptera, Blattodea, Mantodea, Diptera, Hemiptera, Orthoptera, Lepidoptera and Hymenoptera are killed by chilling rather than freezing^35^, suggesting that overwintering temperatures could play a similarly important role in these species. Mechanistic approaches like ours should therefore be useful for a wide range of species and help develop pro-active pest management in a changing world, reduce costs of control efforts, and assure food security while minimizing impacts on natural enemies and other aspects of the ecosystem. In practice, our results emphasize the importance of adjusting pest management strategies to adapt to differences in winter survival across regions and how this will change under future climate scenarios. For instance, in overwintering regions integrated pest management should be especially effective, because it involves non-chemical approaches, or spraying based on economic threshold to avoid unnecessary pesticide application. In case of inevitable spraying, pesticides with very different lethal mechanisms should be alternated to reduce the selection pressure. In non-overwintering regions, we should instead apply different pesticides in the immigrated region from the possible emigration area to reduce the pesticide resistance development.

## Methods

### Insect preparation

We collected 200–300 larvae and pupae of the diamondback moth from cabbage and cauliflower fields in Wuhan, Beijing, and Shenyang in late September from 2008 to 2012. We mixed all collections into one stock colony because of no geographic differentiation in this species from these sites^59^. We reared all individuals on cabbage leaves spread evenly across five screen cages (35 × 35 × 15 cm) in growth chambers at constant temperature (25 ± 1 °C) with 15-h light:9-h dark photoperiod, and relative humidity set at 60 ± 10%. We moved any new pupae to new screen cages for adult emergence. Emerged adults were fed with 10% honey solution in cotton balls. To collect eggs, we dipped four small pieces of laboratory film (7 × 5 cm) in fresh cabbage juice for 3–5 s and hung the treated film pieces on the top of each screen cage. To further enlarge the population for our experiments, we reared these insects in the artificial diet in plastic boxes at 25 ± 1 °C. We transferred 200 eggs to the surface of 120 g artificial diet (Southland Products Incorporated, USA) in each plastic box (10 × 10 × 9 cm). The hatched larvae dropped to the surface of the artificial diet and fed on it. Once individuals developed into 3rd or 4th instar larvae, pupae or adults, they were exposed to 10 °C for 24 h (to simulate gradually reduced temperatures in late autumn and allow a thermal acclimation) just before they were placed to the low-temperature regimes for overwintering tests. Overall, we obtained >7000 larvae and >8000 pupae for the laboratory experiment, and >8000 larvae, >8000 pupae, >4000 adults for the field experiment. We have compared the life history traits of insects reared on the artificial diet with natural host plants (cabbage leaves), they performed similarly (Peng and Li, unpublished data).

### Laboratory experiment of winter survival

#### Site selection

To identify what factor determines winter survival under different winter thermal conditions, we conducted a laboratory experiment that simulated temperature regimes of 10 selected sites across a latitudinal gradient in China (Fig. 1, Supplementary Table 1) at which this species is known to occur and damage cruciferous crops during the growing season.

#### Temperature treatment

To simulate the winter temperatures in the 10 geographically distinct sites (Fig. 1a, Supplementary Table 1), we collected daily mean temperatures during winter (November to next April of 1966–2010) at each site from China Meteorological Data Service Centre (http://data.cma.cn/en). Then, we fitted a polynomial model to the temporal changes of winter daily mean temperatures for each site (Fig. 1b). To simplify the logistics of temperature control procedures, we set all temperature regimes in combinations of linear decline, horizontal maintenance and linear increase to mimic the polynomial changes of winter temperatures in the 10 sites, and adjusted temperature every 10 days as needed (Fig. 1c). We controlled the winter temperature changes of the 10 sites with climate chambers (RXZ-280B, Jiangnan Ltd., Ningbo, China) and refrigerators (Royalstar BCD-246GER) according to curves in Fig. 1c.

#### Experimental protocols

We conducted a winter survival experiment with 10 low-temperature regimes (Fig. 1c). We exposed 6050 larvae and 7150 pupae to 10 low-temperature regimes according to the experiment design (Fig. 1c). Then we sampled 55 larvae and 65 pupae every 10 days from each temperature regime resulting in 11 sampling points. Sampled larvae were placed at 25 °C for 1.5 days to observe the survival based on if their body kept fresh green^60^ and appendage moved after touching with a brush^34^. The pupae were placed at 25 °C, RH 70–80% and photoperiod of 16 L:8D for emergence to determine the survival (emergence rate). These samples were not returned to the temperature treatments. Thus, no individual was measured more than once and each sample interval represents an independent observation.

### Field survival experiments across 12 geographic sites

To verify the cold survivals from the laboratory simulation and identify the best predictor under natural conditions, we conducted field experiments to explore the winter survival for multiple years at various geographic sites in China (Fig. 1a, Supplementary Table 1). The diamondback moth overwinters either in remaining cabbage plants or in fallen leaves (post-harvest conditions) in regions without standing cabbage crops in the winter. We tested the winter survival of larvae, pupae and adults in the caged cabbage plants or post-harvest conditions in fallen leaves on the soil surface at each site for 3–4 months. We transferred 30 larvae, 30 pupae or 30 adults from our stock rearing to a cabbage plant in the field. Then each plant was covered with a screen cage to avoid disturbance and contain focal individuals (see Supplementary Fig. 2). We set 6–8 cages for larvae, pupae and adults, respectively, in a field in November or early December. After an exposure of 1, 2, 3 and 4 months, we collected 2 cages of larvae, 2 cages of pupae and 2 cages of adults from the field at each sampling point and kept individuals in the laboratory (25 ± 1 °C, RH 65–75%, L:D = 16:8 h) for two days. We checked the survival status of the larvae based on the change in body coloration (i.e. if the larval body kept fresh green colour)^60^, pupal survival based on whether adults could emerge from the pupae, and adult survival based on if their appendage moved after touching with a brush.

To simulate the field microenvironment of post-harvest conditions in winter, we filled half of a glass jar (diameter = 5.5 cm, height = 14 cm) with moist soil. Then, we transferred 30 larvae or 30 pupae to the soil surface, covered the insects with leaves, and then covered the glass jar with a nylon net (see Supplementary Fig. 2). We buried 6–8 jars for larvae and pupae, respectively, and kept the top of the jar at ground surface level at each site in November and early December. Because almost all adults died in few days within the jar, we did not test the adult survival in post-harvest conditions. After an exposure of 1, 2, 3 and 4 months, we took 2 jars of larvae and 2 jars of pupae per sampling period from the field and placed them in the laboratory with 25 ± 1 °C, RH 65–75%, L:D = 16:8 h for 2 days. The survival status of the larvae and pupae was checked with the same procedures as the overwintering tests on caged cabbage plants. Note that as in the standing plant experiment, no individual was tested more than once assuming that each observation is independent at the replicate level.

### Modelling and predicting winter survival

#### Model development

Our goal was to identify key metrics that best predict the winter survival of the diamondback moth across a climatic gradient. To achieve this goal, we took several steps. First, we fit a set of predictive models to the laboratory experiments to identify which metric and model best describes survival under controlled conditions. We focused on three alternative predictors: the lowest daily mean temperature (MinDT_mean_), mean temperature (DT_mean_) combined with exposure days, and low-temperature degree-days (LTDD). The MinDT_mean_ model assumes that survival can simply be predicted as a function of the lowest temperature an individual experienced during its exposure time. The DT_mean_ model assumes that survival depends on both the average temperature individuals experience below the cold threshold for survival (11 °C)^32^ and exposure duration (note that exposure time varied systematically in 10-day increments). Finally, the LTDD model predicts survival depending on coldness below the cold threshold. We calculated LTDD by summing up negative deviations of daily mean temperatures from the cold threshold (11.0 °C) during each exposure period for each simulated geographic site (Fig. 1c). To detect potential relationships, we fit each model using three different functions, i.e. linear, exponential and sigmoid models to describe the survival probability (Supplementary Table 2, Fig. 1). We estimated parameters of models in SigmaStat 3.5 and compared model fit using *R*^2^ and AIC values (see detailed models in Supplementary Table 2).

#### Field validation of survival models

To validate winter survival models derived from the laboratory (see models in Supplementary Table 2) for complex and variable field conditions, we compared model predictions to observed survivals in field experiments across 12 different geographic sites over 5 years (Fig. 1a, Supplementary Table 1). To make the connection, we first collected daily mean temperatures recorded at the nearest weather stations to our field sites from China Meteorological Data Service Centre. We then calculated MinDT_mean_, DT_mean_ and exposure days, and LTDD for each site for each treated period and input these values into these laboratory models to predict winter survival. Note, that because the coefficients were calculated from the laboratory experiment, predictions are completely independent of survival observed under field conditions. During the model validation, we excluded the field data of south China, e.g. Guangzhou, Changsha and Wuhan where the warmer temperatures allowed moths to continue their regular life cycle during the whole winter, resulting in unrealistic winter survival. We also excluded replicates in which glass jars were filled with water and destroyed the tested insects. We used linear regression to compare predicted survival with field observations. The validity of each model was evaluated based on the variance explained, slopes of linear regressions and prediction bias (i.e. deviation from unity slope). Finally, we selected the exponential model driven by LTDD as the model to predict the global distribution of winter survival due to its lowest AIC value (Supplementary Table 2) and the least bias (Supplementary Table 3, Fig. 2) among all models.

### Global prediction of overwintering range shift

To extrapolate our winter survival predictions to a global scale under present and future climate conditions, we downloaded global historical daily mean temperature data for 50 years (1967–2016) from Berkeley Earth (1° × 1° grid, http://berkeleyearth.org/data/). We added 1, 2, 3, 4, 5 and 6 °C to mean temperatures of 2012–2016, respectively, to represent the different future warming scenarios^37^. Then, we calculated the annual LTDD in the northern hemisphere with Eq. (1) and in the southern hemisphere with Eq. (2). For *x*_*i,j*_ < *x*_0_,

1 $${{{{{\rm{LTDD}}}}}}={\sum }_{j=182}^{365}|{x}_{i,j}-{x}_{0}|+{\sum }_{j=1}^{181}|{x}_{i+1,j}-{x}_{0}|$$

2 $${{{{{\rm{LTDD}}}}}}\,={\sum }_{j=1}^{365}|{x}_{i,j}-{x}_{0}|$$

where *x*_*i,j*_ is the daily mean temperature at each grid, *i* is the year, *j* is the Julian day, and *x*_0_ = 11.0 °C is the cold threshold for the diamondback moth survival. If *x*_*i,j*_ > *x*_0_, we excluded the *x*_*i,j*_ for the calculation LTDD. For Eq. (1), we started the calculation of LTDD from July 1st (Julian date 182), ended on June 30th of next year (Julian date 181) to cover the whole low-temperature season in the northern hemisphere cross the calendar year. We used LTDD for every year during past conditions to our validated survival model (LTDD-dependent exponential model) and further calculated the expected corresponding yearly winter survival and 5-year mean survival. Since the diamondback moth only feeds on Brassicaceae plants^61^, we incorporated host availability to refine the pest distributions. We retrieved Brassicaceae occurrence data during 1967–2016 (3,720,971 records) from the Global Biodiversity Information Facility (GBIF) database (www.gbif.org), and excluded unknown and duplicate records; 919,808 records were retained to model the global distribution of host plants. We used a dataset of eight selected bioclimatic variables as described in a previous Brassicaceae biogeographic study^62^, including isothermality (bio3), temperature seasonality (bio4), min temperature of coldest month (bio6), mean temperature of wettest quarter (bio8), mean temperature of driest quarter (bio9), precipitation seasonality (bio15), precipitation of warmest quarter (bio18), precipitation of coldest quarter (bio19) from Worldclim dataset^63^ (http://worldclim.org). We ran the species distribution model using the Maxent algorithm in R package *dismo*^64^. Model outputs were presented in grid ranks of host plant presence probability from 0 (unsuitable) to 1 (most suitable). Based on the known distribution of Brassicaceae, we only included grid cells with Brassicaceae presence probability ≥0.3 for our final survival and distribution analysis to ensure the presence of the host plant and mapped them with Arcmap 10.2 (Environmental Systems Research Institute) (see Fig. 3a, e). To show spatial-temporal changes in the geographic distribution of winter survival, we quantified the historical change (expansions or contractions) in the overwintering range based on the total numbers of grids for each year between 1967 and 2016 relative to the baseline area in 1967 (see Fig. 3f) and further calculated average changes of every 5 years (see Fig. 3b). We selected sites in the overwintering marginal belt (with winter survival between 1 and 5%) in the baseline year (1967), calculated the annual LTDD of these sites from 1967 to 2016, and built the linear trend of annual LTDD for years 1967–2016 (see Fig. 3g). We predicted distribution changes for future scenarios (added 1, 2, 3, 4, 5 and 6 °C to the current mean temperatures of 2012–2016) relative to the baseline area of 1967–1971 (see Fig. 3c, d).

### Meta-analysis linking pesticide resistance to overwintering type

#### Data preparation: literature search and selection criteria

We performed a comprehensive literature survey to collect data on pesticide resistance of the diamondback moth worldwide. We searched for publications in databases of ISI Web of Science, Scopus and China National Knowledge Infrastructure (CNKI) using keywords “pesticide resistance” in combination with “diamondback moth” or *“Plutella xylostella”* and expanded references in the selected papers. We reviewed titles, abstracts and in many cases the full articles for relevance and agreement with our inclusion criteria. Studies were included if they (1) monitored the pesticide resistance of field populations, (2) used the leaf dip bioassay method to test pesticide resistance which is the most commonly used method recommended by Insecticide Resistance Action Committee (IRAC, http://www.irac-online.org); (3) provided resistance ratio of field populations. Resistance ratio (abbreviated as RR) is the magnitude of pesticide resistance and is commonly calculated by dividing the median lethal concentration (LC50) of a tested field population by LC50 of the susceptible population (without exposure to pesticide). The LC50 is commonly estimated from a concentration-mortality curve of a given pesticide. The preliminary literature search resulted in 2151 studies out of which 62 matched these criteria. A PRISMA diagram describing details of our literature search is available in Supplementary Fig. 4.

#### Data preparation: data extraction

We extracted data from each selected publication, the names of pesticides, sampling locations and years of field populations, number of tested individuals in a bioassay, resistance ratio of field populations (RR), LC50 of field populations (LC50_field_) and susceptible populations (LC50_susceptible_), and 95% confidence intervals (CIs) of LC50_field_ and LC50_susceptible_. Some studies generated results from multiple types of pesticides with the same field population, each of which was considered as a different entry. Finally, we gathered 1806 entries for pesticide resistance of field populations of the diamondback moth.

#### Data preparation: calculation of the weighted effect size

We conducted a meta-analysis to test if pesticide resistance levels vary across different types of overwintering sites. To account for differences in sample sizes and variances in resistance ratios across studies, we calculated the corrected (weighted) resistance ratio for each study following the method in Hedges et al.^65^. We calculated the logarithm of resistance ratio (logRR) to present the effect size for each entry and further calculated the weighted effect size (wlogRR) by

3 $${{{{{\rm{wlogRR}}}}}}={{{{{\rm{logRR}}}}}}\times w$$

where *w* is the weighting factor of each entry, with *w* = 1/sqrt(V_logRR_)^66^. To consider the contribution from both field and susceptible population, the pooled variance V_logRR_ was calculated as follows^65^:

4 $${{{{{{\rm{V}}}}}}}_{{{{{{\rm{logRR}}}}}}}=\frac{{{{{{{{\rm{SE}}}}}}}_{{{{{{\rm{field}}}}}}}}^{2}}{{n}_{{{{{{\rm{field}}}}}}}\times {{{{{{{\rm{LC50}}}}}}}_{{{{{{\rm{field}}}}}}}}^{2}}+\frac{{{{{{{{\rm{SE}}}}}}}_{{{{{{\rm{susceptible}}}}}}}}^{2}}{{n}_{{{{{{\rm{susceptible}}}}}}}\times {{{{{{{\rm{LC50}}}}}}}_{{{{{{\rm{susceptible}}}}}}}}^{2}}$$

where LC50_field_ and LC50_susceptible_, SE_field_ and SE_susceptible_, *n*_field_ and *n*_susceptible_, are LC50, the standard error of LC50 and sample size for field population and susceptible population, respectively. SE_field_ and SE_susceptible_ can be calculated from their own confidence intervals (95% CI)^67^:

5 $${{{{{\rm{SE}}}}}}=\frac{{{{{{{\rm{CI}}}}}}}_{{{{{{\rm{upper}}}}}}{{{{{\rm{limit}}}}}}}-{{{{{{\rm{CI}}}}}}}_{{{{{{\rm{lower}}}}}}{{{{{\rm{limit}}}}}}}}{2\times 1.96}$$

where CI_upper limit_ is the upper limit and CI_lower limit_ is the lower limit of the 95% CI for LC50.

We used the prognostic method^68^ to estimate V_logRR_ for entries that miss either 95% CI or LC50 based on the average V_logRR_ of the other complete entries.

#### Data preparation: potential moderator variables

Several factors could influence pesticide resistance besides overwintering temperatures. The effective temperature degree-days (ETDD) may change the annual number of generations, the intensity of pesticide application, and thus the selection stress^47^, e.g. between 7.4 and 33 °C for the diamondback moth^32^. In addition, the variety of pesticides used in a study may also affect the resistance levels through their mode of actions (the lethal mechanism) and cross-resistance^69,70^. To account for these potentially confounding factors, we collected the mode of action for each variety of pesticides from IRAC, and calculated LTDD, ETDD and overwintering type for each of the 1806 original records. We collected data for daily mean temperatures for each site from Berkeley Earth. For each location, we calculated the mean annual LTDD, ETDD and winter survival average across the 5 years before the sample. We split the sampling sites into three types based on predicted winter survival of the diamondback moth: (1) the permanent (overwintering) sites: locations with the mean winter survivals ≥5%, (2) marginal sites: locations with the mean winter survivals 1–5%, (3) transient (non-overwintering) sites: locations with the mean winter survivals <1%. We mapped all three category sites in a world map with Arcmap 10.2 (see Fig. 4d).

#### Data analysis: impact of overwintering types

To isolate the effects of overwintering types (permanent, marginal, transient) on resistance from ETDD and pesticide variety, we fit a linear mixed model using *lmer* function from R package *lme4*. We used this model with the weighted effect size (wlogRR) as the response variable, overwintering type and pesticide variety as fixed factors, ETDD as a covariate, and combination of sampling location and year as a random factor. We used a Wald Chi-square test to evaluate the significance of each fixed factor, covariate and their interactions using *Anova* function from R package *car*. R scripts are provided as Supplementary Software.

To show the resistance level in clearer biological meanings, the resistance ratios (RR) were classified into five levels^71^, (1) susceptible: RR ≤ 1, (2) tolerance/low resistance: 1 < RR < 10, (3) moderate resistance:10 ≤ RR < 100, (4) high resistance: 100 ≤ RR < 1000, (5) extremely high resistance: RR ≥ 1000. We calculated the incidence frequencies of these five resistance levels, respectively, and showed the frequencies in a pie chart for the three overwintering types (see Fig. 4a). Furthermore, we calculated the weighted mean resistance ratio^66^ for the permanent, marginal and transient overwintering sites, respectively.

#### Data analysis: distribution of high-level resistance

Pesticide resistance varies markedly from region to region due to different histories of insecticide application on the farm level, which could obscure the differences in statistics between different regions. However, practitioners of pest control are especially concerned about high levels of pesticide resistance in their regions. Thus, we built quantile regression models driven by LTDD to predict the global distribution of resistance levels (log_10_-transformed resistance ratio) in R package *quantreg* (see Supplementary Fig. 3), especially we focused on the significant 0.85 quantile model (see Fig. 4b) for the distribution of the top 15% resistance levels in China and the world in 2012–2016. Areas with no host plants have no resistance problems, so we excluded the grids where Brassicaceae were absent. For each grid cell of the top 15% resistance level, we kept the resistance level as the value if the Brassicaceae presence probability was ≥0.3, otherwise setting it 0. Then we excluded grids with 0 value and mapped with Arcmap 10.2 (see Fig. 4c, d). R scripts are provided as Supplementary Software.

#### Publication bias

We checked for publication bias by drawing a funnel plot of the effect size (logRR) vs. study size (sample size in bioassay) and tested this potential bias statistically using Kendall’s rank correlation^72^. We found no indication for a publication bias (see details in Supplementary Methods for publication bias assessment).

Statistical analyses were done in R v4.0.5 and RStudio 1.1.463.

### Ethics statement

All applicable international, national, and/or institutional guidelines for the care and use of animals were followed. All procedures performed in studies involving animals were in accordance with the ethical standards or practice of the Institute of plant protection, Chinese Academy of Agriculture Sciences, Beijing, China.

### Reporting summary

Further information on research design is available in the Nature Research Reporting Summary linked to this article.

## Supplementary information

Supplementary Information

Description of Additional Supplementary Files

Supplementary Software

Reporting Summary

## Data Availability

The survival data and meta-analysis data in this study have been deposited in the figshare repository at 10.6084/m9.figshare.15052299.v2. All climatic data are available from web databases including China Meteorological Data Service Centre (http://data.cma.cn/en) by registering an account, Berkeley Earth (http://berkeleyearth.org/data/), and WorldClim (http://www.worldclim.org). Brassicaceae plants occurrence data are freely accessible in Global Biodiversity Information Facility (http://www.gbif.org/). Source data are provided with this paper.

## Source data

Source data

## Code availability

The main R functions and packages used in this study are provided in ‘Methods’. Full R scripts are provided as Supplementary Software.
