# Supplementary material for: Climate warming promotes pesticide resistance through expanding overwintering range of a global pest
Source: Nat Commun. 2021 Sep 9;12:5351. doi: 10.1038/s41467-021-25505-7 (PMC8429752; doi:10.1038/s41467-021-25505-7)
Supplement: Supplementary file 1 — Supplementary Information [file 41467_2021_25505_MOESM1_ESM.pdf]

Supplementary Information for

Climate warming promotes pesticide resistance through expanding overwintering range of a global pest

Chun-Sen Ma*, Wei Zhang*, Yu Peng, Fei Zhao, Xiang-Qian Chang, Kun Xing, Liang Zhu, Gang Ma, He-Ping Yang, Volker H.W. Rudolf

*Corresponding author: Chun-Sen Ma, Wei Zhang

Email: machunsen@caas.cn, zhangwei06@caas.cn

This PDF file includes:

Supplementary Figs 1 to 4
Supplementary Tables 1 to 3
Supplementary Methods
Supplementary References

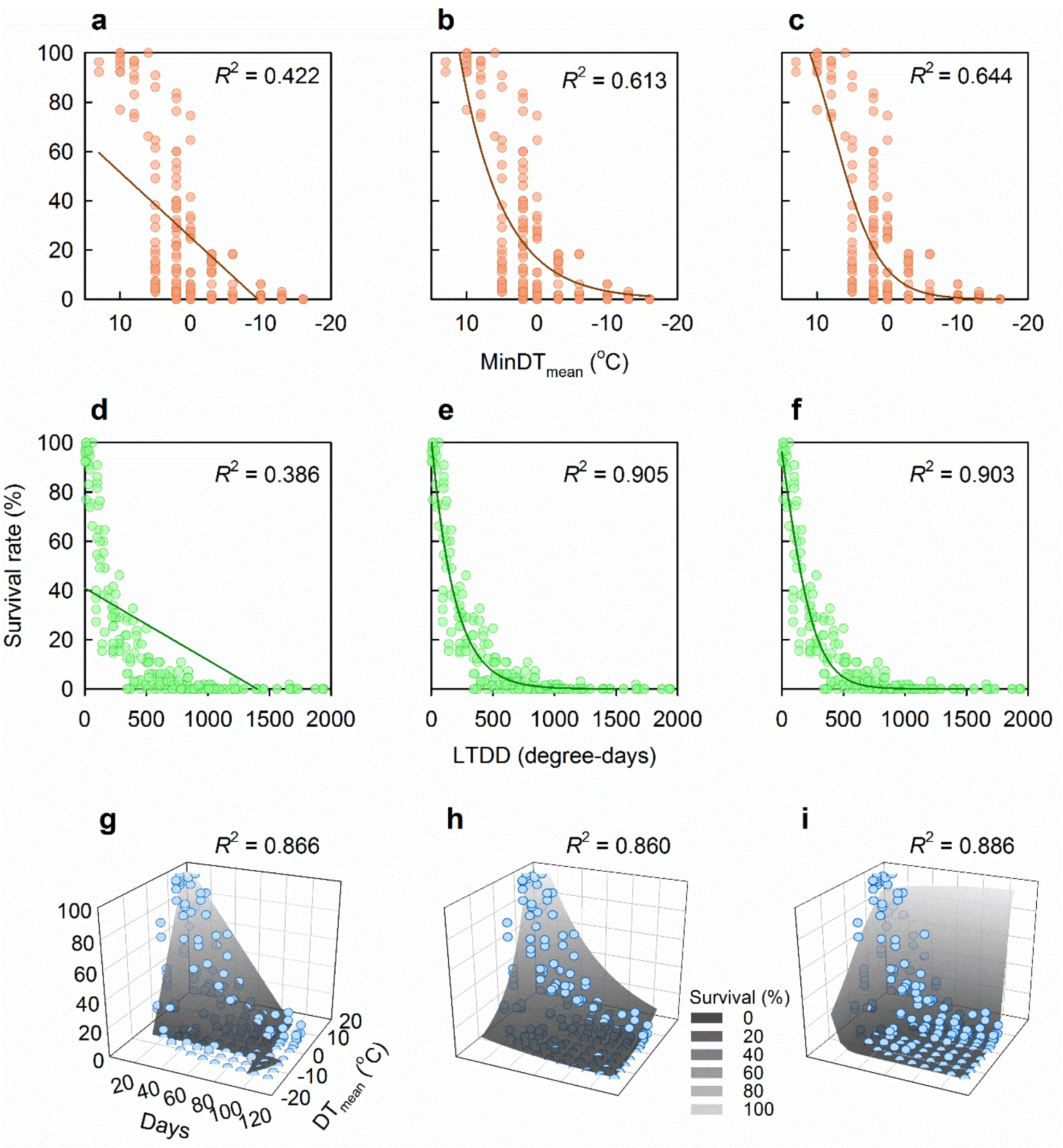

Supplementary Fig. 1. Survival rate as a function of low winter temperatures. Three panels in the same row represent linear, exponential and sigmoid models respectively. (a-i) represent models (see Supplementary Table 2) with three different predictors: lowest daily mean temperatures ($MinDT_{mean}$) (a-c), low temperature degree-days (LTDD) (d-f), and mean temperature and exposure days (DT_{mean}×Time) (g-i). Solid circles (*n* = 220) are the observed survival rates at different thermal conditions in the laboratory and solid lines (a-c, d-f) or surfaces (g-i) represent survival predictions of different models. Source data are provided as a Source Data file.

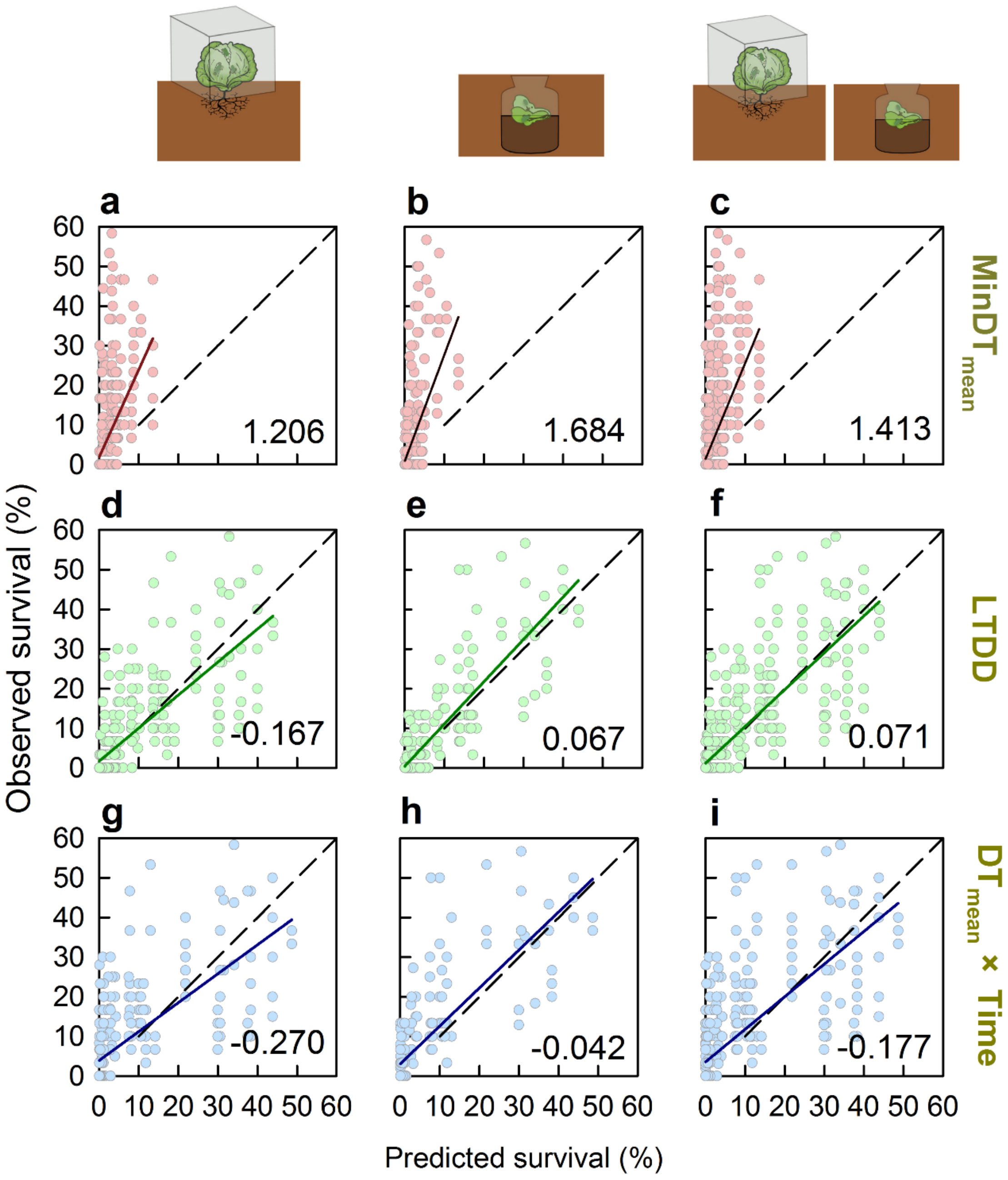

Supplementary Fig. 2. Winter survival model validations. (a-i) represent the best fit models for three independent predictors: lowest daily mean temperature ($MinDT_{mean}$), low temperature degree-days (LTDD), and mean temperature and exposures days (DT_{mean}×Time) (see Supplementary Table 2). Solid circles represent observed survivals in caged standing plants (n = 322), in gaps of post-harvest residues (n = 234) and in both (n = 556). Lines are linear regressions between predictions and observed survivals in caged standing plants, in gaps of post-harvest residues and in both. The dashed line of each panel indicates the 1:1 reference line. The value on the lower right corner of each panel is the bias of slope for prediction from the observed survivals. Source data are provided as a Source Data file.

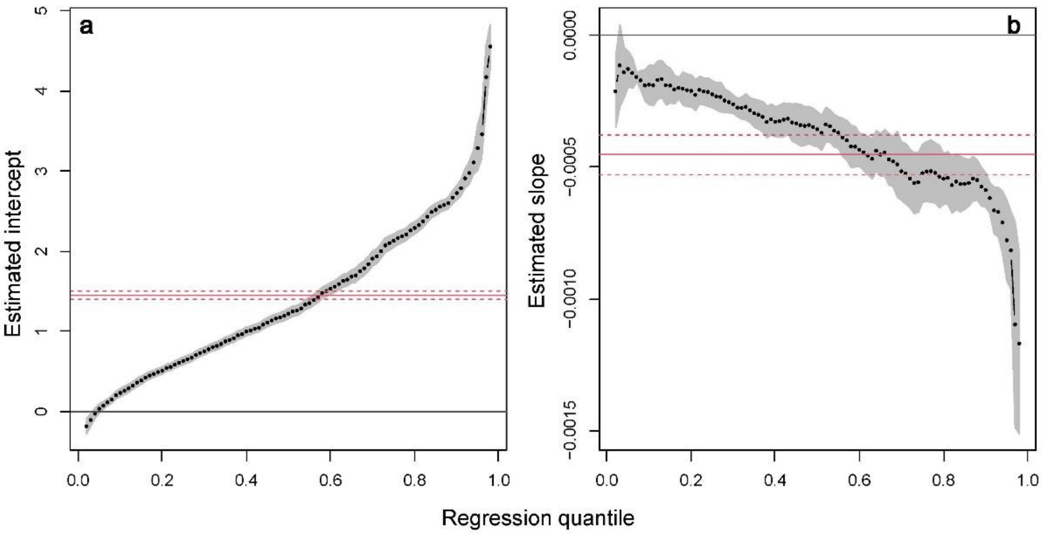

Supplementary Fig. 3. Estimated coefficients from the quantile linear regression models between low temperature degree-days (LTDD) and logarithm of resistance ratios. (a) shows the intercepts and (b) shows the slopes of predicted linear models at different quantile levels. Shaded area indicates bootstrapped 95% confidence intervals. The red solid lines show the intercept and slope estimated by the ordinary least squares method for the linear relationship between LTDD and logarithm of resistance ratios, with 95% confidence intervals (red dashed lines). Source data are provided as a Source Data file.

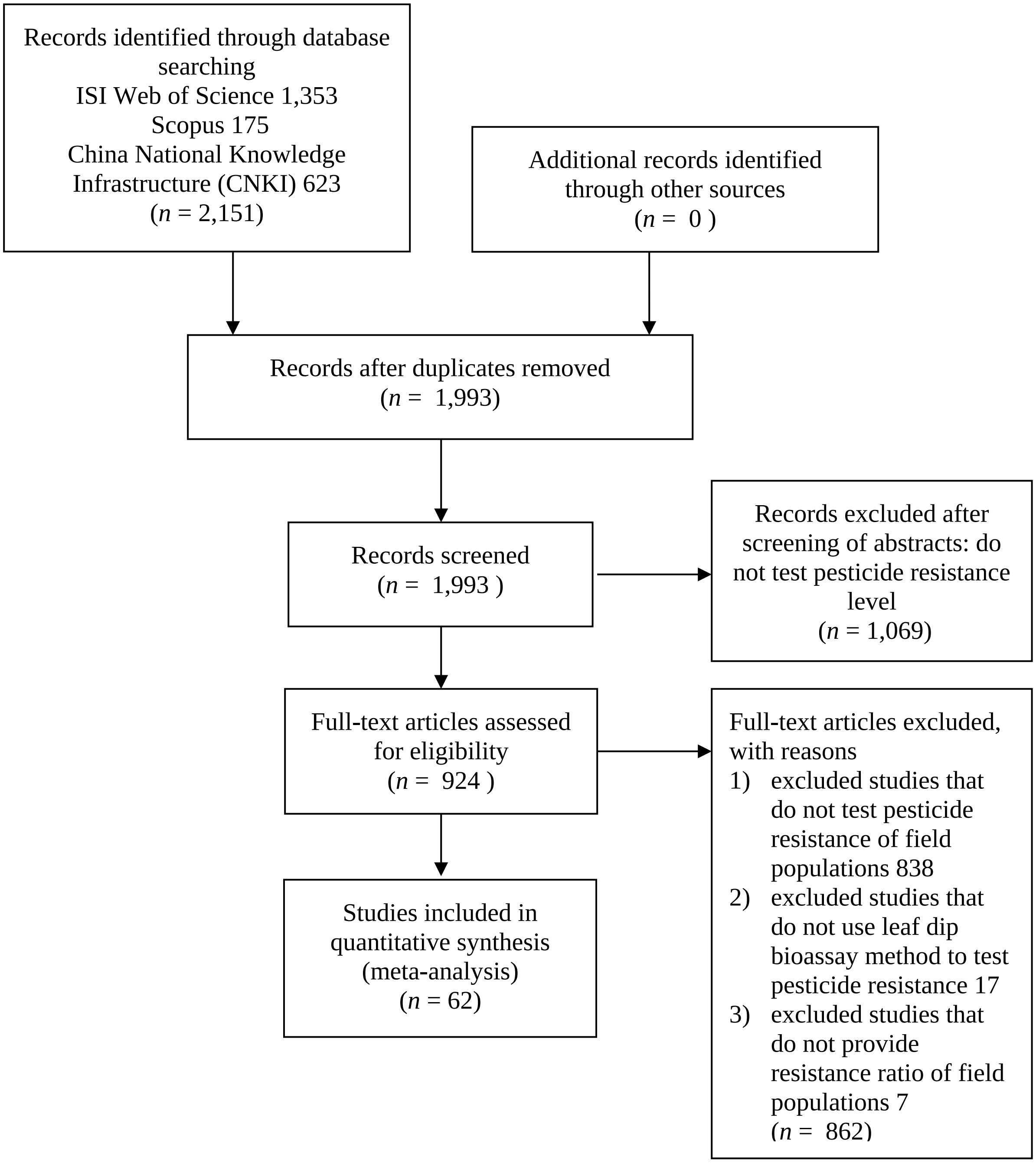

Supplementary Fig. 4. PRISMA diagram. Flow of inclusion and exclusion of studies identified during the literature search, presented as a PRISMA diagram with number of published papers in brackets.

Supplementary Table 1. Experiment sites and years to test overwintering survival of the diamondback moth.

Experimental sites	Latitude	Longitude	Altitude (m)	Mean daily mean (1966-2015) (°C)	Mean daily max (1966-2015) (°C)	Mean daily min (1966-2015) (°C)	Experimental winter (November-April)				
							2008-2009	2009-2010	2010-2011	2011-2012	2012-2013
Harbin(HRB)*	45°45'N	126°46'E	146.0	-7.9	-2.2	-13.4					
Siping(SP)*	43°11'N	124°20'E	164.2	-4.5	1.3	-9.6					
Shenyang(SY)*	41°46'N	123°26'E	41.6	-2.5	3.4	-7.8	Y				
Datong(DT)	40°06'N	113°20'E	1067.6	-2.9	4.3	-9.1		Y	Y		
Beijing(BJ)*	39°35'N	116°19'E	29.4	3.2	8.8	-1.8	Y	Y			
Yinchuan(YC)*	38°31'N	106°16'E	1111.0	0.0	7.2	-4.5					
Shijiazhuang(SJZ)*	38°03'N	114°26'E	78.0	5.2	11.2	0.5		Y	Y	Y	Y
Taiyuan(TY)	37°47'N	112°33'E	777.9	1.5	8.6	-4.4			Y		
Huimin(HM)	37°49'N	117°51'E	12.0	3.6	9.8	-0.9	Y				
Anyang(AY)*	36°03'N	114°24'E	62.9	5.4	11.2	0.6	Y	Y	Y	Y	Y
Zhengzhou(ZZ)*	34°43'N	113°39'E	110.4	6.4	12.6	2.0		Y	Y	Y	Y
Zhumadian(ZMD)*	33°00'N	114°01'E	82.7	7.1	12.4	2.7	Y	Y	Y		
Wuhan(WH)*	30°62'N	114°08'E	23.1	9.1	13.8	5.5	Y			Y	Y
Changsha(CS)	28°23'N	112°94'E	44.9	9.7	14.9	6.5	Y				
Guangzhou(GZ)	23°13'N	113°26'E	6.6	17.2	21.7	14.0	Y				

Laboratory and field experiments testing overwintering of the diamondback moth under field conditions at different sites and winters (years). * Sites are selected for overwintering experiments in the laboratory. The year interval indicates the respective winter season and “Y” in the column represents that the data were available in that site and the year.

Supplementary Table 2. Winter survival models and estimated parameters.

Independent variable	Regression model	Equation	Parameters	R^2	AIC
	Linear	$z= a_0+ a_1*x$	$a_0 = 25.251$, $P < 0.001$; $a_1 = 2.633$, $P < 0.001$	0.422	1370.277
x = $MinDT_{mean}$	Exponential	$z=a*\exp(b*x)$	$a = 16.720$, $P < 0.001$; $b = 0.162$, $P < 0.001$	0.613	1282.176
	**Sigmoidal**	$\mathbf{z= a/(1+\exp(b*(x-x_0)))}$	**$a = 135.101$, $P < 0.001$; $b = -0.304$, $P < 0.001$; $x_0 = 7.617$, $P < 0.001$**	**0.644**	**1265.571**
	Linear	$z= a_0+ a_1*x$	$a_0 = 40.870$, $P < 0.001$; $a_1 = -0.0292$, $P < 0.001$	0.386	1383.466
x = LTDD	**Exponential**	$\mathbf{z=a*\exp(b*x)}$	**$a = 101.004$, $P < 0.001$; $b = -0.00531$, $P < 0.001$**	**0.905**	**973.141**
	Sigmoidal	$z= a/(1+\exp(b*x))$	$a = 192.504$, $P < 0.001$; $b = 0.00734$, $P < 0.001$	0.903	977.555
	Linear	$z=a_0+a_1*x+a_2*y+a_3*x*y$	$a_0 = 38.185$, $P < 0.001$; $a_1 = 6.206$, $P < 0.001$; $a_2 = -0.391$, $P < 0.001$; $a_3 = -0.0647$, $P < 0.001$	0.866	1052.619
x = DT_{mean}<br>y = Time	Exponential	$z=a*\exp(b*x+c*y)$	$a = 44.395$, $P < 0.001$; $b = 0.103$, $P < 0.001$; $c = - 0.0221$, $P < 0.001$	0.860	1061.075
	**Sigmoidal**	$\mathbf{z=100*\exp(a+b*(x-11)*y) /(1+\exp(a+b*(x-11)*y))}$	**$a = 1.688$, $P < 0.001$; $b = 0.0111$, $P < 0.001$**	**0.886**	**1013.380**

Winter survival models built by survival rate (laboratory results) and independent low temperature predictors [lowest daily mean temperature ($MinDT_{mean}$), low temperature degree-days (LTDD), or mean temperature and exposures (DT_{mean}×Time)]. The significance of regression parameters were tested by two-sided t-test. R^2 indicates the variability explained by the regression model. The Akaike information criterion (AIC), $AIC = 2 \times k + n \times \ln(SSR/n)$, where k is the number of parameters in the model, n is the number of observations used in the model, SSR is the sum of squared residuals. The smaller AIC indicates better goodness of the model fit. Three models in bold are the best fit models for the three independent predictors. To build the sigmoid model of DT_{mean} and time, we consider the time-temperature model in cold injury[1].

Supplementary Table 3. Relationship between model predictions and field observations of winter survival.

Independent variable	Model	Validation for survivorship in caged standing plants	R^2	*P*	Validation for survivorship in gaps of post-harvest residues	R^2	*P*	Validation for total survivorship	R^2	*P*
MinDT$_{mean}$	Linear	$y = 0.373 x + 5.211$	0.117	<0.001	$y = 0.469 x - 4.723$	0.136	<0.001	$y = 0.412 x + 5.008$	0.124	<0.001
	Exponent	$y = 1.537 x - 2.293$	0.180	<0.001	$y = 1.893 x - 4.326$	0.219	<0.001	$y = 1.688 x - 3.172$	0.197	<0.001
	**Sigmoid**	**$y = 2.206 x + 1.913$**	**0.192**	**<0.001**	**$y = 2.684 x + 0.888$**	**0.234**	**<0.001**	**$y = 2.413 x + 1.455$**	**0.210**	**<0.001**
LTDD	Linear	$y = 0.627 x - 3.848$	0.392	<0.001	$y = 0.760 x - 6.277$	0.405	<0.001	$y = 0.680 x - 4.809$	0.395	<0.001
	**Exponent**	**$y = 0.833 x + 1.733$**	**0.572**	**<0.001**	**$y = 1.067 x + 0.432$**	**0.695**	**<0.001**	**$y = 0.929 x + 1.187$**	**0.621**	**<0.001**
	Sigmoid	$y = 0.784 x + 2.818$	0.541	<0.001	$y = 1.016 x + 1.734$	0.673	<0.001	$y = 0.879 x + 2.360$	0.595	<0.001
DT$_{mean}$×Time	Linear	$y = 0.603 x + 0.891$	0.520	<0.001	$y = 0.714 x - 0.146$	0.537	<0.001	$y = 0.648 x + 0.466$	0.524	<0.001
	Exponent	$y = 0.993 x - 4.721$	0.588	<0.001	$y = 1.218 x - 7.219$	0.653	<0.001	$y = 1.085 x - 5.748$	0.613	<0.001
	**Sigmoid**	**$y = 0.730 x + 3.945$**	**0.484**	**<0.001**	**$y = 0.958 x + 3.087$**	**0.618**	**<0.001**	**$y = 0.823 x + 3.580$**	**0.539**	**<0.001**

The equations are linear regressions between field observed survival (*y*) and model predicted survival (*x*). The closer 1 the slope is, the more accurately the model predicted. The significance of linear regression (*P* value) was tested by two-sided Fisher's test. R^2 indicates the variability explained by the model. Three models in bold are the best fit models for the three independent predictors.

Supplementary Methods. Assessment of potential publication bias

Effect size vs. sample size. We estimated precision based on sample size. Note that proportion of sample size for field population and susceptible population (n_F : n_S） are 1:1 for Non-overwintering region (N) and Permanent-overwintering region (P), but below one for Marginal-overwintering region (M). So, we just show sample size for field population (n_F) here.

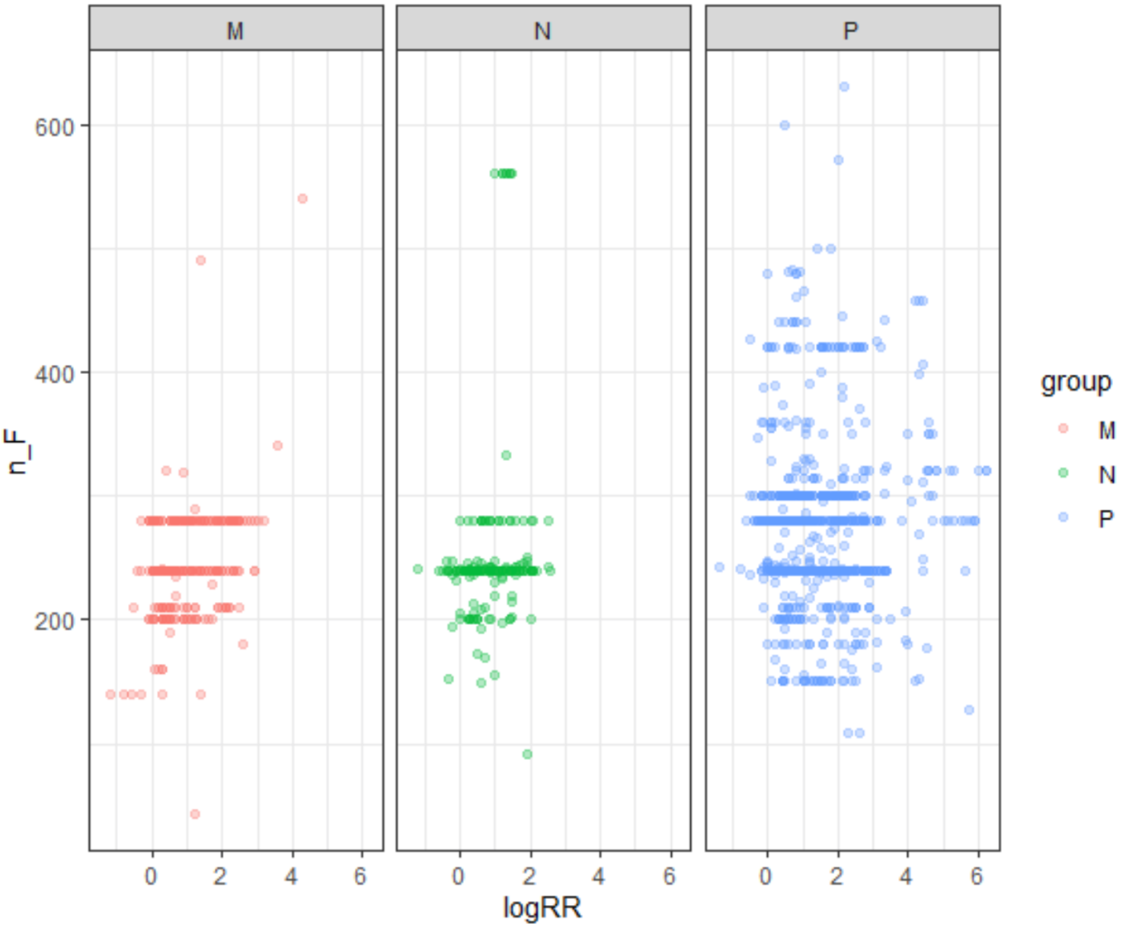

There is no indication for a general publication bias, i.e. low effect sizes are not underrepresented (this would be indicated by fewer studies than expected in the lower left corner) and large effect sizes are not over represented. More importantly, since we are comparing overwintering sites (M vs N vs P), the more crucial and important comparison is whether there are differences in publication bias across sites. Overall, patterns are the same across all sites, so there is no evidence that publication bias differed across sites and influenced our overall conclusions. We tested this potential bias statistically using Kendall's rank correlation[2]. A significant negative correlation would indicate a bias. We found no significant correlation in Marginal-overwintering sites ($\tau = -0.000018$, $P = 0.999$) and significant positive correlation in Non-overwintering sites ($\tau = 0.139$, $P < 0.001$) and

Permanent-overwintering sites ($\tau = 0.196$, $P < 0.001$). Overall, this indicates that there was no publication bias in any site type and if anything, our results are slightly conservative.

Supplementary References

1. Nedvěd, O., Lavy, D. & Verhoef, H. A. Modelling the time–temperature relationship in cold injury and effect of high-temperature interruptions on survival in a chill-sensitive collembolan. *Funct. Ecol.* **12**, 816-824 (1998).
2. Begg, C. B., Mazumdar M. Operating characteristics of a rank correlation test for publication bias. *Biometrics* **50**, 1088-1101 (1994).
