# Supplementary material for: Climate warming promotes pesticide resistance through expanding overwintering range of a global pest
Source: Nat Commun. 2021 Sep 9;12:5351. doi: 10.1038/s41467-021-25505-7 (PMC8429752; doi:10.1038/s41467-021-25505-7)
Supplement: Supplementary file 2 — Description of Additional Supplementary Files [file 41467_2021_25505_MOESM2_ESM.pdf]

Description of Additional Supplementary Files

File name: Supplementary Software 1

Description: R language code used to build host plant global distribution, quantile regression analysis, linear mixed model analysis and Wald Chi-square test in this study.
