# Supplementary material for: Climate warming promotes pesticide resistance through expanding overwintering range of a global pest
Source: Nat Commun. 2021 Sep 9;12:5351. doi: 10.1038/s41467-021-25505-7 (PMC8429752; doi:10.1038/s41467-021-25505-7)
Supplement: Supplementary file 4 — Reporting Summary [file 41467_2021_25505_MOESM4_ESM.pdf]

nature research

Corresponding author(s): Chun-Sen Ma and Wei Zhang

Last updated by author(s): Jul 26, 2021

Reporting Summary

Nature Research wishes to improve the reproducibility of the work that we publish. This form provides structure for consistency and transparency in reporting. For further information on Nature Research policies, see our Editorial Policies and the Editorial Policy Checklist.

Statistics

For all statistical analyses, confirm that the following items are present in the figure legend, table legend, main text, or Methods section.

n/a	Confirmed	
☐	☒	The exact sample size (n) for each experimental group/condition, given as a discrete number and unit of measurement
☐	☒	A statement on whether measurements were taken from distinct samples or whether the same sample was measured repeatedly
☐	☒	The statistical test(s) used AND whether they are one- or two-sided<br>*Only common tests should be described solely by name; describe more complex techniques in the Methods section.*
☐	☒	A description of all covariates tested
☒	☐	A description of any assumptions or corrections, such as tests of normality and adjustment for multiple comparisons
☐	☒	A full description of the statistical parameters including central tendency (e.g. means) or other basic estimates (e.g. regression coefficient) AND variation (e.g. standard deviation) or associated estimates of uncertainty (e.g. confidence intervals)
☐	☒	For null hypothesis testing, the test statistic (e.g. F, t, r) with confidence intervals, effect sizes, degrees of freedom and P value noted<br>*Give P values as exact values whenever suitable.*
☒	☐	For Bayesian analysis, information on the choice of priors and Markov chain Monte Carlo settings
☒	☐	For hierarchical and complex designs, identification of the appropriate level for tests and full reporting of outcomes
☐	☒	Estimates of effect sizes (e.g. Cohen's d, Pearson's r), indicating how they were calculated

Our web collection on statistics for biologists contains articles on many of the points above.

Software and code

Policy information about availability of computer code

Data collection	No computer code was used to collect the data in this study.
Data analysis	Parameters of cold survival models were estimated in SigmaStat v3.5. Host plant global distribution was predicted using the Maxent algorithm in R package "dismo". Mixed linear model was done using lmer function from R package "lme4". Wald Chi-square test was done using Anova function from R package "car".Quantile regression analysis was done in R package "quantreg". These statistical analyses were done in R v4.0.5 and RStudio 1.1.463. All R scripts are provided as Supplementary software.

For manuscripts utilizing custom algorithms or software that are central to the research but not yet described in published literature, software must be made available to editors and reviewers. We strongly encourage code deposition in a community repository (e.g. GitHub). See the Nature Research guidelines for submitting code & software for further information.

Data

Policy information about availability of data

All manuscripts must include a data availability statement. This statement should provide the following information, where applicable:

- Accession codes, unique identifiers, or web links for publicly available datasets
- A list of figures that have associated raw data
- A description of any restrictions on data availability

Data supporting the findings of this study are provided in the publicly accessible repository, public web databases and a supplementary file for Source data. The survival data and meta-analysis data have been deposited in the figshare repository at https://doi.org/10.6084/m9.figshare.15052299.v2. All climatic data are available from web databases including China Meteorological Data Service Centre (http://data.cma.cn/en) by registering an account, Berkeley Earth (http://berkeleyearth.org/data/), and WorldClim (http://www.worldclim.org). Brassicaceae plants occurrence data are freely accessible in Global Biodiversity Information Facility (http://www.gbif.org/). The source data underlying Figs 1-4 and Supplementary Figs 1-3 are provided as Source data file.

Field-specific reporting

Please select the one below that is the best fit for your research. If you are not sure, read the appropriate sections before making your selection.

- [ ] Life sciences
- [ ] Behavioural & social sciences
- [x] Ecological, evolutionary & environmental sciences

For a reference copy of the document with all sections, see nature.com/documents/nr-reporting-summary-flat.pdf

Ecological, evolutionary & environmental sciences study design

All studies must disclose on these points even when the disclosure is negative.

Study description	We combined field and laboratory experiments with global climate modelling and a global meta-analysis to explore that overwintering survival drives the evolution of pesticide resistance of a global destructive pest, the diamondback moth. Laboratory survival experiment was conducted with 10 temperature regimes representing different sites in China (Fig. 1 and Supplementary Table 1). Field survival tests were performed in winter in 2008-2013 at 12 various geographic sites in China (Fig. 1a and Supplementary Table 1). We built climate models by fitting 9 models to the laboratory experiments and validating the models by comparing model predictions to observed survivals in the field tests. We performed a comprehensive literature survey to collect data on global pesticide resistance of the diamondback moth and linked the pesticide resistance level with overwintering survival and winter coldness.
Research sample	Sampling was performed to maximize sample size afforded time, personnel, and resources, including 13,200 individuals in laboratory test and 17,563 individuals of the diamondback moth (Plutella xylostella) in field test. In laboratory test, we exposed 13,200 individuals to 10 temperature regimes and 11 exposure days, 120 individuals /(regime*exposure). In field test, we exposed 17,563 individuals to 12 geographic sites and 4 exposure months, >360 individuals/(site*exposure month). These samples represent the populations of the diamondback moth which have the potential to experience low temperatures in the winter. We collected 1,806 insecticide resistance records of Plutella xylostella from 62 published papers according to our criteria described in PRISMA (see Supplementary Fig. 4 PRISMA diagram). These records represent the populations of the diamondback moth in the world that pesticide resistance were potentially impacted by overwintering range.
Sampling strategy	We don’t fully understand what the sampling strategy exactly is. Indeed, we followed certain principles during sampling in experiments. For laboratory and field studies, we randomly and evenly assigned individuals to each treatment, and then tested the survival status of all individuals. In the meta-analysis, we searched for publications in databases of ISI Web of Science, Scopus and China National Knowledge Infrastructure (CNKI) using keywords “pesticide resistance” in combination with “diamondback moth” or “Plutella xylostella” and expanded references in the selected papers. We reviewed titles, abstracts and in many cases the full articles for relevance and agreement with our inclusion criteria. Studies were included if they: (1) monitored the pesticide resistance of field populations, (2) used the leaf dip bioassay method to test pesticide resistance which is the most commonly used method recommended by Insecticide Resistance Action Committee (IRAC, http://www.irac-online.org); (3) provided resistance ratio of field populations. A PRISMA diagram describing details of our literature search is available in Supplementary Fig. S4. This resulted in 62 papers, with a total of 1806 records.
Data collection	X.Q.C., K.X., G.M. and H.P.Y. collected survival data using pen and paper by checking the survival status of the larvae based on the change in body coloration and if their appendage moved after touching with a brush, pupal survival based on whether adults could emerge from the pupae, and adult survival based on if their appendage moved after touching with a brush. C.S.M and W.Z. performed a comprehensive literature survey and extracted data from each selected publication (including the names of pesticides, sampling locations and years of field populations, number of tested individuals in a bioassay, resistance ratio of field populations, LC50 of field populations and susceptible populations and 95% confidence intervals of LC50).
Timing and spatial scale	In laboratory experiments, we sampled every 10 days at the same time after the beginning of the experiment and stopped on 110 days at this timing all tested individuals either completely died or survivors could continue their regular life cycle. In the field survey we also found 110-day cold exposure is long enough to test the low temperature survival. In field experiments, we collected data after an exposure of 1, 2, 3 and 4 months in winter from 2008-2013 at 12 geographic sites in China. Details of experiment sites are provided in the Supplementary Table 1. We chose these sites because: 1) diamondback moth seriously damages local cabbage, 2) we got the permission to do field work in these sites, and 3) these sites located in north and south of China could create different winter conditions for field survival experiments. The experiments were conducted on post-harvest conditions, and start time in each site was depend on harvest time (November-December). Based on the knowledge of field population dynamics of diamondback moth, we stopped the experiments after 4 months (March-April) because at that time the moths have appeared in fields of marginal overwintering belt while the moths completely die in cold non-overwintering area. We performed a literature survey of publications since 1980s and collected 1,806 published global insecticide resistance records.
Data exclusions	No lab survival data were excluded from the modeling fitting. Field survival data at Guangzhou, Changsha and Wuhan were excluded from model validation, because warmer temperatures of these sites allowed moths to continue their regular life cycle during the whole winter, resulting in unrealistic winter survival. We also excluded replicates in which glass jars were filled with water and destroyed the tested insects. These exclusion criteria were not pre-established. In the meta-analysis, we excluded data based on pre-established exclusion criteria listed in the methods and Supplementary Fig. 4 PRISMA diagram.
Reproducibility	All laboratory and field studies were completed once, but used maximal sample sizes afforded time, personnel, and resources. All raw data has been provided in order to facilitate replication of statistical analyses. Not relevant for the meta-analysis.
Randomization	For laboratory and field studies, samples were randomly assigned to treatment groups prior to treatment. Not relevant for the meta-analysis.

Blinding	Blinding is not relevant to this study, because the data collectors are unaware of data analysis. Not relevant for the meta-analysis.

Did the study involve field work? ☒ Yes ☐ No

Field work, collection and transport

Field conditions	Field studies were conducted in winter (November-April) of 2008-2013 at 12 various geographic sites in China. Further descriptions of field conditions, i.e. experimental sites, Daily Mean temperature (1966-2015), Daily Max temperature (1966-2015), Daily Min temperature (1966-2015) and experiments years can be found in the Supplementary Table 1.
Location	Field studies were conducted at 12 various geographic sites in China, and latitudes, longitudes, altitudes of these sites were provided in the Fig. 1 and Supplementary Table 1.
Access & import/export	Fieldwork permits were issued from 2008 to 2013 by Plant Protection Agency in Shenyang, Beijing, Shijiazhuang, Datong, Taiyuan, Huimin, Anyang, Zhengzhou, Zhumadian, Wuhan, Changsha, and Guangzhou.
Disturbance	Field work were conducted after harvest, and researchers entered sites on foot causing minimal disturbance.

Reporting for specific materials, systems and methods

We require information from authors about some types of materials, experimental systems and methods used in many studies. Here, indicate whether each material, system or method listed is relevant to your study. If you are not sure if a list item applies to your research, read the appropriate section before selecting a response.

Materials & experimental systems

n/a	Involved in the study
☒	☐ Antibodies
☒	☐ Eukaryotic cell lines
☒	☐ Palaeontology and archaeology
☒	☐ Animals and other organisms
☒	☐ Human research participants
☒	☐ Clinical data
☒	☐ Dual use research of concern

Methods

n/a	Involved in the study
☒	☐ ChIP-seq
☒	☐ Flow cytometry
☒	☐ MRI-based neuroimaging
